# Functional Characterization of the *PAX8* p.Leu264Pro Variant Identified in a Patient with a Müllerian Duct Anomaly

**DOI:** 10.3390/genes17091039

**Published:** 2026-08-29

**Authors:** Lin He, Liangzhe Li, Yuxiao Li, Yujun Sun, Zhi Zheng, Chunfang Chu, Lin Li

**Affiliations:** 1 Central Laboratory, Beijing Obstetrics and Gynecology Hospital, Capital Medical University, Beijing Maternal and Child Health Care Hospital, Dongcheng, Beijing 100006, China; 2 Department of Gynecology, Beijing Obstetrics and Gynecology Hospital, Capital Medical University, Beijing Maternal and Child Health Care Hospital, Chaoyang, Beijing 100026, China

**Keywords:** Müllerian duct anomaly, *PAX8*, whole-exome sequencing, RNA-seq

## Abstract

Background/Objectives: Müllerian duct anomalies (MDAs) are congenital structural abnormalities of the female reproductive tract with heterogeneous and incompletely defined genetic contributions. *PAX8* is a developmental transcription factor implicated in thyroid and urogenital development. This study assessed the functional consequences of a rare *PAX8* variant identified by whole-exome sequencing (WES) in an MDA cohort, without presuming a causal relationship. Methods: WES was performed in 150 patients with MDAs. The *PAX8* c.791T>C (p.Leu264Pro) variant was evaluated using computational predictions, cellular assays, and RNA sequencing in a 293FT transient-overexpression model. Results: The heterozygous p.Leu264Pro variant was identified in one patient with a complex septate uterine, cervical, and vaginal anomaly and was absent from 120 controls. Parental samples were unavailable. No statistically significant difference in construct-derived *PAX8* mRNA abundance was detected between PAX8-WT and PAX8-L264P, and both proteins showed predominantly nuclear localization. In the direct PAX8-WT-versus-L264P RNA-seq comparison, 76 transcripts had nominal *p* values below 0.05, but none remained significant after Benjamini–Hochberg correction, and no gene met the prespecified differential-expression criteria. *AK5*, *RCBTB2*, and *IFT88* were identified as candidate *PAX8*-responsive genes in this experimental system. Conclusions: No major functional difference was detected between *PAX8*-WT and L264P under the tested conditions; however, these findings do not establish functional equivalence or exclude smaller, tissue-specific, or developmental-stage-specific effects. The p.Leu264Pro variant remains a variant of uncertain significance with respect to MDAs, and the functional results were not used as benign evidence under ACMG/AMP criterion BS3.

## 1. Introduction

Müllerian duct anomalies (MDAs) refer to a group of developmental malformations affecting the female genital tract, resulting in a spectrum of reproductive abnormalities. They are observed in approximately 6.7% of the general female population and in 16.7% of women with a history of recurrent miscarriages [1]. MDAs can cause symptoms such as dysmenorrhea, dyspareunia, and impaired fertility, negatively affecting both physical and mental health [2]. According to the ASRM Müllerian anomalies classification 2021 [3], the clinical manifestations of MDAs are highly variable, spanning from isolated defects within a single reproductive organ to dysgenesis involving multiple organs, and they may also coexist with abnormalities in other systems or with various syndromes. Most affected individuals have a normal karyotype, but a normal karyotype does not exclude sequence-level variants or submicroscopic copy-number changes.

The genetic architecture of MDAs is heterogeneous. Unraveling the precise etiology of MDAs remains a formidable challenge, largely attributable to the intricate and highly dynamic nature of female reproductive tract embryogenesis, which is governed by a multifaceted interplay of genetic, epigenetic, and environmental factors. The advent of high-throughput sequencing technologies has markedly accelerated the discovery of numerous candidate susceptibility genes, including *TBX6*, *PAX8*, *BMP4*, *BMP7*, *HOXA10*, *EMX2*, *LHX1*, *WNT9B*, *WNT4*, *GREB1L*, *HNF1B*, *OSR1*, *NR6A1*, *CHD1L*, *HOXD10*, and *AR* [4,5,6,7,8,9,10,11,12,13,14], thereby providing valuable insights into the genetic etiology of MDAs. However, most candidate genes cannot fully explain these anomalies [15,16]. Several factors contribute to this limitation, including modest sample sizes, functional redundancy among paralogous genes, complex genotype–phenotype correlations, and gene–environment interactions. Furthermore, the incomplete and variable penetrance characteristic of MDAs often results in inconsistent genotype–phenotype associations, wherein identical genomic aberrations can manifest as markedly disparate clinical presentations—a phenomenon suggestive of a genetic architecture far more intricate than classical Mendelian inheritance [17].

The PAX (paired box) domain family, a group of developmental transcription factors, consists of nine members that play a role in the differentiation of various organs [18]. Numerous genes are expressed in the embryonic urogenital system, including *PAX8*, and play important roles during embryonic urogenital development [19,20]. Also, the *PAX8* gene is essential for the development of the thyroid [20], kidneys [21], and Müllerian duct derivatives [22]. Throughout the entire development of the Müllerian ducts (MDs), from the initial stages through to adulthood, *PAX8* is consistently expressed and plays a direct morphogenic role in uterine development. Although *Pax8*-deficient mice display a severely reduced uterine muscle layer and no endometrial tissue, the fallopian tubes, cervix, and vagina develop without any apparent abnormality [22], suggesting that other transcription factors compensate for the absence of *PAX8* in these structures [20].

A study of 422 individuals with Mayer–Rokitansky–Küster–Hauser (MRKH) syndrome found a higher mutational rate of *PAX8* in MRKH patients compared to healthy controls, and an association with congenital hypothyroidism was noted [5]. Heterozygous variants or deletions of the *PAX8* gene have been identified in patients with congenital hypothyroidism combined with urogenital malformations [23,24]. Collectively, these findings suggest that *PAX8* may play a pathogenic role in MDAs, particularly in patients presenting with concurrent hypothyroidism.

Despite these observations, the contribution of *PAX8* variants to MDAs remains incompletely understood. We identified the rare p.Leu264Pro variant in one patient and assessed its functional consequences without presuming a causal relationship. The resulting model-specific evidence informs interpretation of this variant but does not establish pathogenicity or benignity. *AK5*, *RCBTB2*, and *IFT88* were identified as candidate *PAX8*-responsive genes in this experimental system rather than established direct transcriptional targets.

## 2. Materials and Methods

### 2.1. Patients and Ethical Approval, WES, In Silico Analysis, and Sanger Sequencing

This study enrolled patients diagnosed with MDAs at Beijing Obstetrics and Gynecology Hospital, Capital Medical University, from October 2018 to April 2026. Detailed methodology was described in our previous study [14]. Genomic DNA extraction and Sanger sequencing were performed following a previously described protocol [13]. Whole-exome sequencing (WES) libraries were prepared using the Agilent SureSelect Human All Exon V6 kit (Agilent Technologies, Santa Clara, CA, USA) according to the manufacturer’s instructions. WES was performed by Annoroad Genomics (Beijing, China) and Novogene Bioinformatics Technology Co., Ltd. on the Illumina NovaSeq 6000 platform, with paired-end 150 bp (PE150) reads generated for each sample. Raw sequencing reads were aligned to the human reference genome GRCh37/hg19. Variants, including single-nucleotide polymorphisms (SNPs) and small insertions/deletions (indels), were called using the Genome Analysis Toolkit (GATK) and subsequently annotated with ANNOVAR (**v2020-06-07**). The potential functional impact of each variant (deleterious versus benign) was predicted using three complementary algorithms: PolyPhen-2, SIFT, and MutationTaster. Variants were filtered to retain only those classified as missense, nonsense, frameshift, or splice-site altering and with a minor allele frequency (MAF) of <1%, based on data from the Genome Aggregation Database (gnomAD), the NHLBI Exome Sequencing Project (ESP6500), and the 1000 Genomes Project (1000G). Sanger sequencing was carried out with locus-specific primers (listed in Table A1) to verify the variant identified by WES.

### 2.2. Plasmid Construction and Transfection

Human *PAX8* wild-type (WT, NM_003466) pcDNA3.1(+)-CMV-*PAX8*-3×Flag and mutant (c.791T>C:p.L264P) overexpression plasmids were purchased commercially from Ruibiotech (Beijing, China), with the empty pcDNA3.1(+) vector used as a control. Transient transfections were carried out using jetPRIME^®^ reagent (Polyplus, Illkirch-Graffenstaden, France, Cat# 101000046) according to the manufacturer’s protocol.

### 2.3. Cell Culture and Reagents

The culture of 293FT cells was performed using Dulbecco’s Modified Eagle’s Medium (DMEM, Thermo Fisher Scientific, Waltham, MA, USA, Cat# CT11995500BT) supplemented with 10% fetal bovine serum (FBS; ExCell, Shanghai, China Cat# FSP500), 1× penicillin–streptomycin ((Thermo Fisher Scientific, Waltham, MA, USA, Cat# 15070063), 1× GlutaMAX™-I ((Thermo Fisher Scientific, Waltham, MA, USA, Cat# 35050079), and 1× MEM Non-Essential Amino Acids (NEAA; Thermo Fisher Scientific, Waltham, MA, USA, Cat# 11140050). Incubation was carried out at 37 °C in a humidified incubator with 5% CO_2_.

### 2.4. Antibodies

The related primary antibodies included those against the GAPDH antibody (60004-1-Ig; ProteinTech, Wuhan, China) and the DYKDDDDK tag antibody (66008-4-Ig; ProteinTech, China). The secondary antibodies used were anti-mouse IgG (H+L) biotinylated antibody (ZB-2305/ZB-2301; ZSGB-BIO, Beijing, China) and anti-mouse IgG (H+L) cross-adsorbed secondary antibody Alexa Fluor™ 488 (A-11005; Invitrogen, Carlsbad, CA, USA). Antibodies were used with appropriate dilutions conforming to standard protocols.

### 2.5. RNA Extraction, Reverse Transcription, Real-Time Quantitative Polymerase Chain Reaction (qPCR), RNA Sequencing (RNA-Seq), and Data Analysis

RNA extraction, cDNA synthesis, and quantitative real-time PCR (qPCR) assays were performed following a previously described protocol [25]. Gene expression levels were normalized against the endogenous control ACTB and analyzed via the 2^−ΔΔCt^ method for relative quantification. All primer sequences are provided in Table A2.

Total RNA was extracted as described above, and its quality was assessed using an Agilent 2100 Bioanalyzer (Agilent Technologies, Palo Alto, CA, USA). RNA-seq libraries were constructed and sequenced on the Illumina NovaSeq 6000 platform (San Diego, CA, USA) by Novogene (Beijing, China), with three biological replicates per sample.

For library preparation, polyadenylated mRNA was enriched using Oligo(dT)-conjugated magnetic beads, then chemically fragmented into short segments. First-strand cDNA was synthesized from the fragmented mRNA using reverse transcriptase, followed by second-strand synthesis upon addition of buffer, dNTPs, and DNA polymerase. The resulting double-stranded cDNA was purified, subjected to end-repair, A-tailing, and adapter ligation, and subsequently size-selected. Libraries were then enriched via PCR amplification. Initial quantification was performed with a Qubit 3.0 fluorometer, and accurate library concentrations were determined by quantitative real-time PCR (qPCR).

Raw sequencing reads were quality-filtered to obtain clean, high-quality reads for downstream gene expression and structural analyses. Differentially expressed genes (DEGs) were defined as those with |log2(Fold Change)| ≥ 1 and adjusted *p*-value (*p*adj) < 0.05. Gene Ontology (GO) enrichment analysis was performed using Metascape (http://metascape.org/gp/index.html, accessed on 25 April 2026), a comprehensive gene annotation and analysis resource.

### 2.6. Immunofluorescence Staining

293FT cells were seeded at an appropriate density and transfected with the respective *PAX8* plasmids. Detailed methodology was described in our previous study [25].

### 2.7. Statistical Analysis

Data are presented as mean ± standard deviation (SD) from at least three independent experiments (biological replicates). Differences among multiple groups were analyzed using a one-way analysis of variance. Significance levels were defined as follows: *** *p* < 0.001, ** *p* < 0.01, * *p* < 0.05, and ns = not significant.

## 3. Results

### 3.1. Clinical Profiles of the MDA Patient Harboring the PAX8 Variant p.Leu264Pro

**Figure 1:**
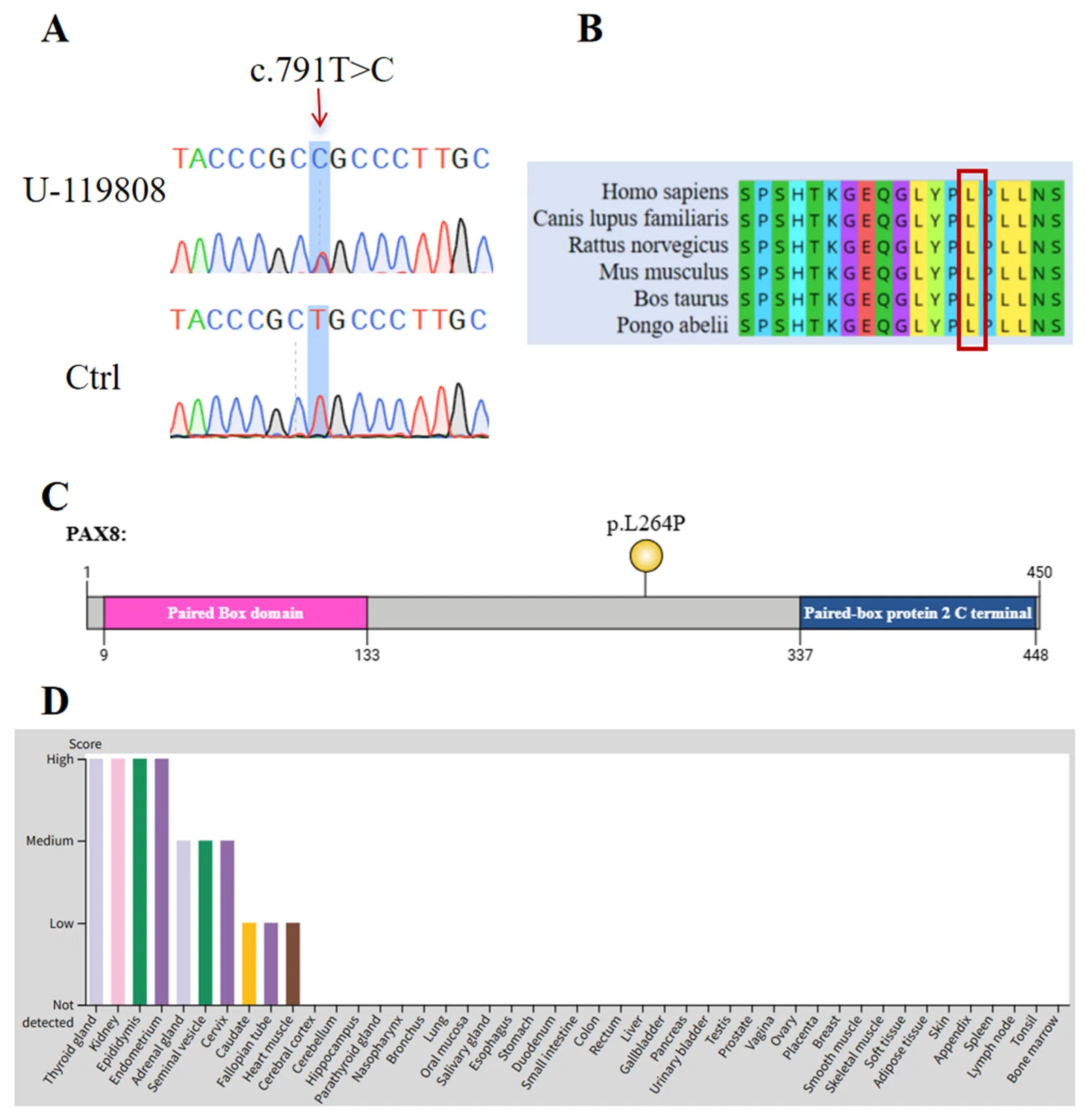
Genetic analysis of the *PAX8* variant: (**A**) Sanger sequencing confirmed the variant in patient U-119808. The red arrow indicates the mutational site (c.791T>C). (**B**) The leucine residue at position 264 (L264) is evolutionarily conserved across multiple species. The affected residue L264 was marked by red rectangle. (**C**) Domain architecture of the PAX8 protein and the position of the L264P variant. PAX8 protein length, 450 aa; Pax2_C domain, residues 337–448. (**D**) Gene expression for *PAX8*. The data were obtained from an online database (https://www.proteinatlas.org/ENSG00000125618-PAX8/tissue, accessed on 5 May 2026) (view ENSG00000125618 on the GTEx website).

A total of 150 Chinese Han patients diagnosed with Müllerian duct anomalies (MDAs) treated at our hospital were enrolled in this study, along with 120 unaffected individuals as controls. Whole-exome sequencing (WES) revealed that one patient carried a novel heterozygous missense variant in the *PAX8* gene (NM_003466.4), specifically c.791T>C (NP_003457.1:p.Leu264Pro, abbreviated as L264P), which was absent in the WES of the control group. This finding was initially confirmed by Sanger sequencing (Figure 1A). No other consistent pathogenic variants associated with MDAs were identified. The *PAX8* c.791T>C (p.Leu264Pro) variant was present in the heterozygous state in the proband. Parental samples were unavailable; therefore, segregation analysis could not be performed, and it remains unknown whether the variant was inherited or arose de novo. Detailed clinical characteristics of patients and in silico prediction results are summarized in Table 1 and Table 2. The available renal and urinary tract evaluation showed no abnormalities. No thyroid function test results or thyroid imaging data were available; therefore, the presence or absence of a thyroid abnormality could not be determined. Computational predictions are presented as supporting evidence of pathogenicity. The L264P-affected amino acid residue is evolutionarily conserved across species (Figure 1B). Notably, this variant is extremely rare in public databases, with only 10 alleles observed in gnomAD v4 (allele frequency = 6.36 × 10^−6^) and no homozygous individuals, and it has not been previously reported in disease-specific databases such as ClinVar. The position of the mutant L264P is illustrated in Figure 1C. On PAX8 (450 aa), NCBI CDD annotates the paired-box domain at residues 9–133 and the Pax2_C conserved C-terminal domain at residues 337–448; Leu264 lies between these annotated domains. According to The Protein Atlas, PAX8 is strongly expressed in the thyroid gland, fallopian tube, cervix, and kidney (Figure 1D). Therefore, functional analyses are warranted to further assess its pathogenicity. Using the ACMG/AMP framework with ClinGen refinements, PM2_Supporting was applied because of the extremely low population frequency of the variant, and PP3_Moderate was applied on the basis of the calibrated REVEL score of 0.773. The results of correlated computational predictors were not counted independently. Parental samples were unavailable; therefore, no de novo or segregation criteria were applied. The absence of an exact ClinVar record was treated as descriptive information and was not used as evidence for classification. BS3 was not applied because the 293FT transient-overexpression assays were not clinically validated and did not model the relevant developmental context. The variant was therefore classified as a variant of uncertain significance with respect to MDAs.

### 3.2. Comparison of PAX8 mRNA Abundance and Subcellular Localization Between WT and L264P

**Figure 2:**
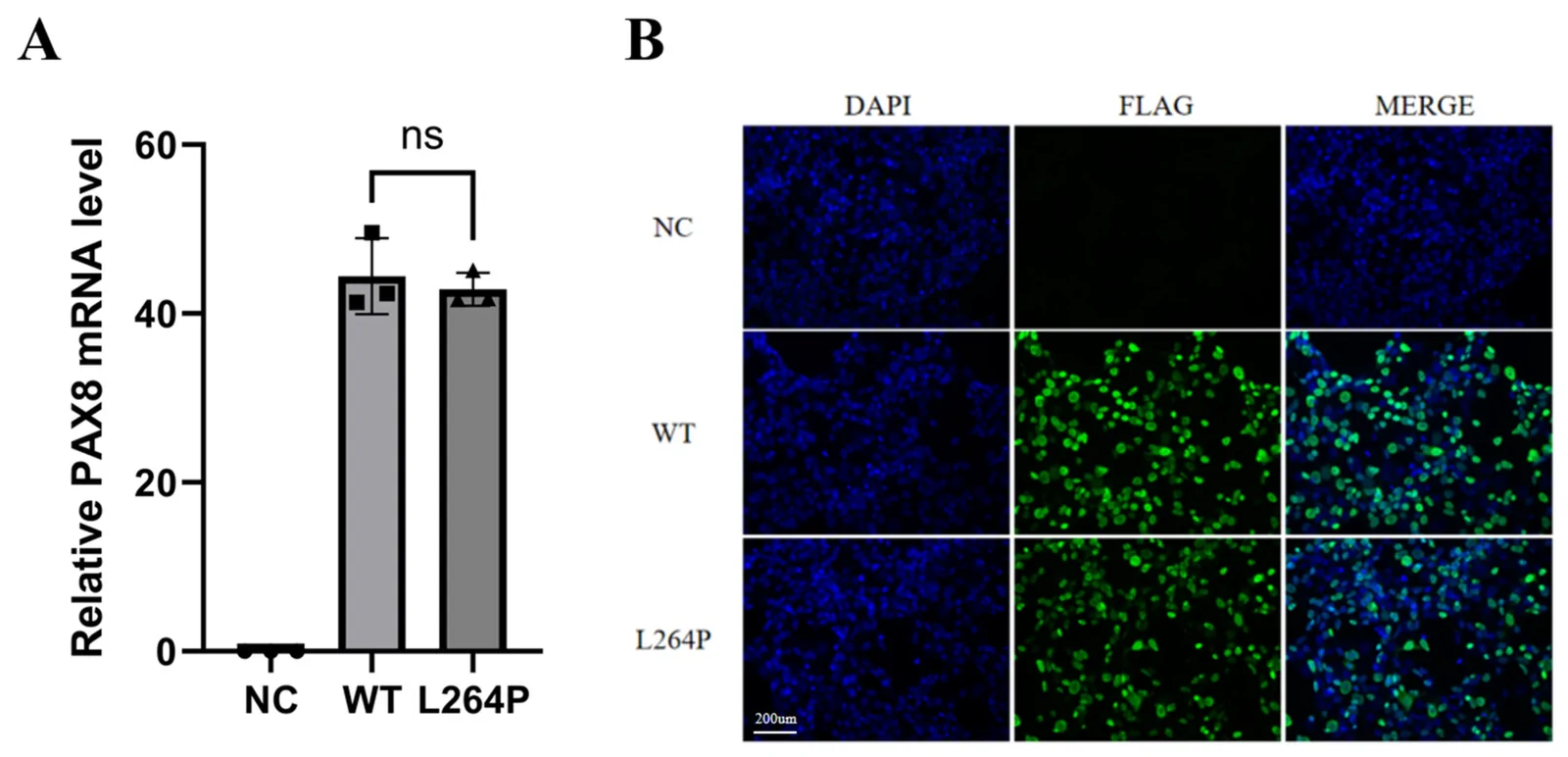
Expression and subcellular localization of PAX8-wild-type (WT) and PAX8-L264P in cells: (**A**) Reverse transcription polymerase chain reaction analyses of the mRNA level of the PAX8 L264P variant. *ACTB* was used as the reference gene. Data are presented as mean ± SD from three independent biological replicates; ns, not significant. (**B**) Immunofluorescence staining of WT and mutant PAX8 proteins. The cellular nucleus was visualized using DAPI (blue). The flag-tagged PAX8 protein (WT or L264P) was stained in green. Scale bar, 200 µm.

To examine the cellular effects of p.Leu264Pro, 293FT cells were transfected with constructs expressing *PAX8*-WT or L264P. No statistically significant difference in construct-derived *PAX8* mRNA abundance was detected between the two groups under the tested conditions (Figure 2A). Immunofluorescence staining showed predominantly nuclear localization of both *PAX8*-WT and L264P, with no apparent qualitative difference in subcellular distribution (Figure 2B). These assays did not directly assess protein abundance or stability and do not provide evidence of benignity.

### 3.3. Transcriptomic Comparison of PAX8-WT and PAX8-L264P

**Figure 3:**
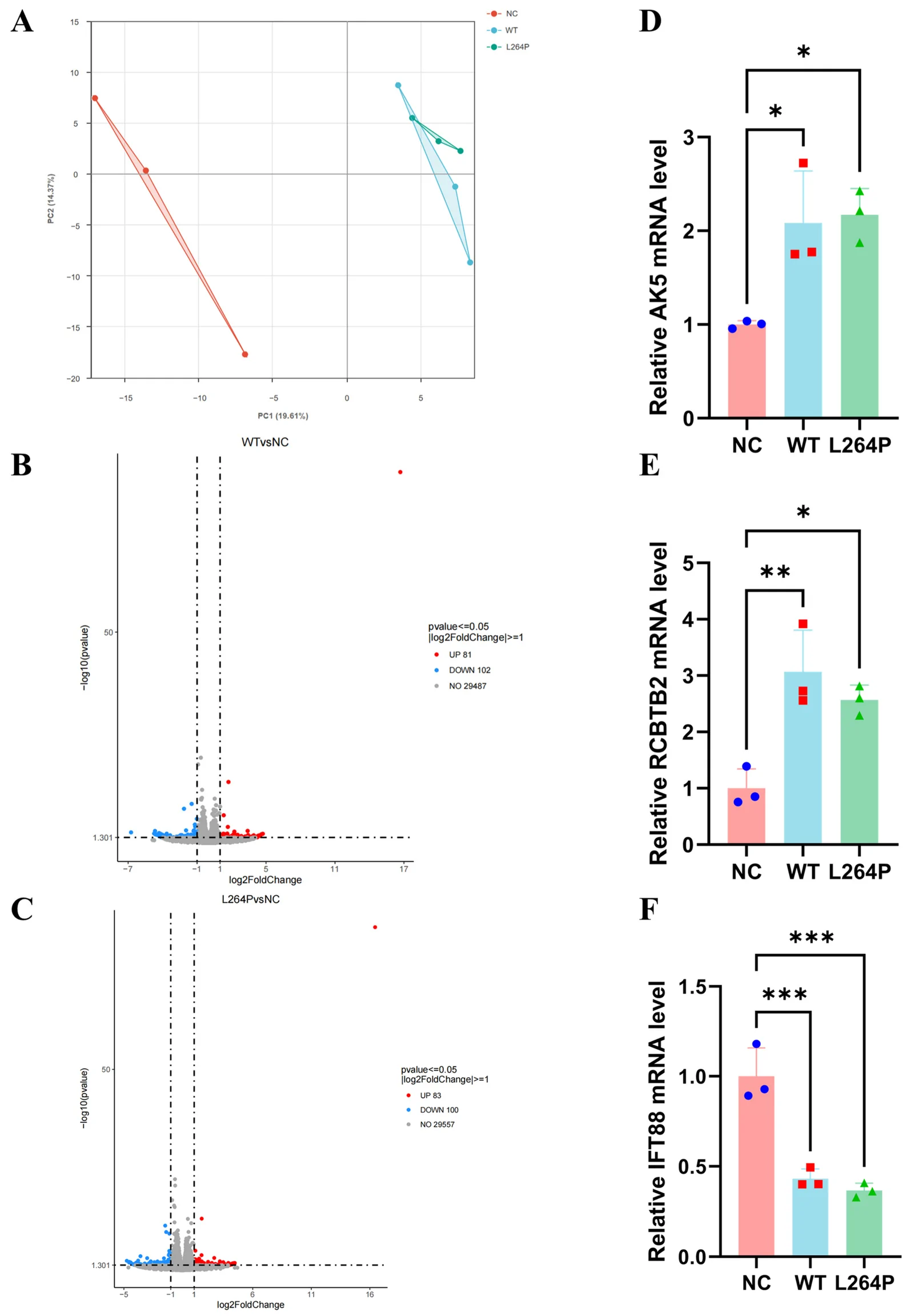
Transcriptomic profiles of cells expressing *PAX8*-WT, *PAX8*-L264P, or the empty-vector control: (**A**) Principal component analysis (PCA) of transcriptome data. Red dots represent the negative control group (NC), blue dots represent the wild-type *PAX8* overexpression group (WT), and green dots represent the *PAX8* L264P variant overexpression group (L264P). (**B**) Volcano plot showing differentially expressed genes (DEGs) between the WT and NC groups. Red dots indicate genes upregulated in the WT group, while blue dots indicate genes downregulated in the WT group. DEGs were defined as genes with |log_2_(fold change)| ≥ 1 and adjusted *p*-value (*p*adj) ≤ 0.05. (**C**) Volcano plot showing DEGs between the L264P and NC groups. Red dots indicate genes upregulated in the L264P group, while blue dots indicate genes downregulated in the L264P group. The DEG filtering criteria were identical to those in panel (**B**). (**D**–**F**) Reverse transcription-quantitative polymerase chain reaction (RT-qPCR) validation of selected DEGs (*AK5*, *RCBTB2*, and *IFT88*). *ACTB* was the reference gene. Different colored dots/squares/triangles mean biological replicate number. The complete unfiltered results for the direct WT-versus-L264P comparison and the corresponding sample-level normalized expression values are provided in Supplementary Tables S1 and S2, respectively. Data in panels (**D**–**F**) are presented as mean ± SD from three biological replicates. *** *p* < 0.001, ** *p* < 0.01, * *p* < 0.05.

RNA sequencing was performed in cells expressing *PAX8*-WT (WT), *PAX8*-L264P (L264P), or the empty-vector negative control (NC), with three biological replicates per condition. The raw RNA-Seq data have been deposited in the NCBI Sequence Read Archive (SRA) under accession number PRJNA1518172. In the PCA plot, the NC samples were separated from samples expressing either *PAX8* construct, whereas the *PAX8*-WT and *PAX8*-L264P samples occupied overlapping regions (Figure 3A). PCA was used as a descriptive visualization of sample-level variation and was not interpreted as a formal test of differential expression.

We next performed a direct transcriptome-wide comparison between the *PAX8*-WT and L264P groups. Although 76 transcripts had nominal *p* values below 0.05, none remained statistically significant after Benjamini–Hochberg correction (adjusted *p* > 0.05 for all tested genes; Supplementary Table S1). Thus, no gene met the prespecified criteria for differential expression in the direct WT-versus-L264P comparison. The complete unfiltered differential-expression results and sample-level normalized expression values for the three WT and three L264P biological replicates are provided in Supplementary Tables S1 and S2, respectively. These results indicate that no transcriptome-wide significant expression differences were detected between *PAX8*-WT and L264P under the experimental conditions and with the sample size used. They do not establish functional equivalence or exclude smaller transcriptional effects.

Given the absence of significant differences between the *PAX8*-WT and L264P groups, we next focused on the differential gene expression profiles between the WT/L264P groups and the NC group. Seven significantly differentially expressed genes (DEGs) with an adjusted *p*-value (*p*adj) < 0.05 were identified between the WT and NC groups (Figure 3B), while 10 significant DEGs (*p*adj < 0.05) were detected between the L264P and NC groups (Figure 3C). Compared with the NC group, seven genes met the prespecified differential-expression criteria in the *PAX8*-WT group, and 10 genes met these criteria in the L264P group. RT-qPCR confirmed the directions of change in *AK5*, *RCBTB2*, and *IFT88* relative to NC (Figure 3D–F). The direction of change was the same in the two PAX8-versus-NC comparisons for these selected genes; however, this observation does not establish equivalent transactivation or transrepression activity between WT and L264P.

## 4. Discussion

The Müllerian ducts originate from the intermediate mesoderm of the urogenital ridge bilaterally during the 6–9.5th embryonic week. Their development into mature, functional female reproductive organs is a dynamic process. Animal models have revealed multiple regulatory factors critical for this process in humans. Due to the close developmental interactions among the Wolffian ducts, Müllerian ducts, and kidneys, MDAs often co-occur with renal or urinary tract anomalies. Nevertheless, several molecular genetic aspects of Müllerian duct development still await clarification.

The *PAX8* gene encodes a paired-box transcription factor that is highly expressed in the Müllerian ducts, Wolffian ducts, and kidney during embryonic development. Along with *PAX2*—a closely related gene that shares partially overlapping functions with *PAX8*—it initiates the mesenchymal–epithelial transition, a process that subsequently leads to the formation of the Wolffian ducts [26]. Through comparative genomic hybridization (CGH) array [22,24], WES [5], and animal experiments [20], many studies have identified *PAX8* as a candidate gene for MDAs. Smol et al. [22] described an additional patient with a 2q12.1q14.1 microdeletion, where *PAX8* is located, in MRKH and congenital hypothyroidism due to thyroid gland hypoplasia through CGH. Zhu et al. [5] identified c.136G>A, c.236C>G, c.619C>T, c.195delC, c.25+1G>T, and c.156_157dupCG through WES in patients with MRKH syndrome. Buchert et al. [27] detected a *PAX8* variant (c.1315G>A) in twins discordant for MRKH through WGS and RNA sequencing. In thyroxine-treated *Pax8*-null females, a functional uterus was absent, the vaginal opening was absent, and only remnants of myometrial tissue remained, whereas the oviducts, cervix, and upper vagina were relatively unaffected; hydrosalpinx occurred frequently [20]. A fusion protein that includes the PAX8 DNA-binding domain is thought to facilitate cell cycle advancement, reduce apoptotic activity, and boost cell growth [28]. Hence, it was hypothesized that variants in *PAX8* might cause MDAs. The phenotype observed in our patient (vaginal septum) contrasts with the *Pax8* knockout mouse model (no vaginal defect) and the typical MRKH presentations, further supporting that this specific missense change might represent a rare polymorphism or a modifier rather than a monogenic cause in this particular case.

Across the cellular and transcriptomic assays, no statistically significant difference in construct-derived *PAX8* mRNA abundance, subcellular localization, or FDR-adjusted gene expression was detected between WT and L264P under the tested conditions. These negative findings do not establish functional equivalence and cannot exclude smaller effects that were beyond the power or biological scope of the present experiments. In particular, the transient-overexpression system in 293FT cells does not reproduce the cellular context or developmental timing of Müllerian duct formation.

Relative to the empty-vector control, expression of either *PAX8* construct was associated with increased *AK5* and *RCBTB2* expression and decreased *IFT88* expression. These genes should therefore be considered candidate *PAX8*-responsive genes in this experimental system rather than established direct transcriptional targets. Their potential involvement in Müllerian duct development remains to be determined. No renal or urinary tract abnormality was documented in the available clinical records, whereas thyroid function testing and thyroid imaging data were unavailable. Parental samples were also unavailable. Consequently, phenotypic concordance, segregation, and de novo occurrence could not be fully evaluated. The functional findings were not used to apply BS3 because the assays lacked a physiologically relevant developmental context and clinical validation with independently classified control variants.

This study has several limitations. First, only one individual carried the p.Leu264Pro variant, and parental samples were unavailable, precluding segregation analysis and determination of whether the variant occurred de novo. Second, thyroid phenotyping was incomplete, and the available renal and urinary tract information was limited to the clinical record. Third, the functional experiments used transient overexpression in 293FT cells, which does not reproduce the tissue-specific and developmental context of Müllerian duct formation. Fourth, the RNA-seq analysis included three biological replicates per condition and therefore had limited power to detect smaller transcriptional effects. Future studies should evaluate the variant in developmentally relevant cellular or organoid models and use clinically validated functional assays that include independently classified pathogenic and benign control variants.

## 5. Conclusions

The *PAX8* p.Leu264Pro variant remains a variant of uncertain significance with respect to MDAs. No statistically significant difference between WT and L264P was detected in construct-derived mRNA abundance, subcellular localization, or FDR-adjusted transcriptomic effects under the conditions tested. These findings do not establish functional equivalence or benignity and cannot exclude smaller, tissue-specific, or developmental-stage-specific effects. *AK5*, *RCBTB2*, and *IFT88* are candidate *PAX8*-responsive genes in this experimental system but are not established direct transcriptional targets. Additional segregation, clinical phenotyping, and functionally validated studies in developmentally relevant models will be required to clarify the clinical significance of p.Leu264Pro.

## Figures and Tables

**Table 1:** Baseline information and in silico analysis of the *PAX8* variant identified in the patient with MDAs.

Parameter	Result
Mutation type	Missense
Exon	8
Variant (NM_003466.4)	c.791T>C
Amino acids (NP_003457.1)	p.L264P
Zygosity	Heterozygous
Parental origin	Unknown (parental samples unavailable)
gnomAD v4	Joint AC/AN 10/1,571,682; AF 6.36 × 10^−6^; no homozygotes
ClinVar	No exact record found
AlphaMissense	0.3936 (Ambiguous)
REVEL	0.773
CADD	26.6 (Likely deleterious)
FATHMM-MKL	Uncertain
MutationTaster	Deleterious
PolyPhen-2	Possibly Damaging
PROVEAN	−3.13 (Deleterious)
SNPs&GO	Disease
ACMG classification	VUS
ACMG/AMP evidence applied	PM2_Supporting; PP3_Moderate; BS3 not applied

**Table 2:** Clinical profile of the patient with *PAX8* variant.

Parameter	Patient U-119808
Age at diagnosis, years	19
Baseline parity	Nullipara
Diagnosis of MDA	Complex septate uterine, cervical, and vaginal anomaly
Other diagnosis	Pyocolpos
Acién and Acién classification	3A5
VCUAM classification	V2b, C1, U1c, A0, M0
ESHRE/ESGE classification	U2bC1V1
ASRM classification	Septate uterus
Renal/urinary tract findings	No abnormality documented in the available clinical records
Thyroid assessment	Thyroid function and imaging data unavailable

Abbreviations: VCUAM, vagina, cervix, uterus, and adnexa-associated malformation; ESHRE/ESGE, European Society of Human Reproduction and Embryology/European Society for Gynecological Endoscopy; ASRM, American Society for Reproductive Medicine.

## Data Availability

The raw RNA-Seq data have been deposited in the NCBI Sequence Read Archive (SRA) under accession number PRJNA1518172 and persistent link (https://dataview.ncbi.nlm.nih.gov/object/PRJNA1518172?reviewer=f4tl37ok4mlguk55olbdlcd2ba accessed on 5 May 2026). The WES data are available from the corresponding author upon reasonable request, subject to applicable consent and privacy restrictions.

## Supplementary Materials

The following supporting information can be downloaded at https://www.mdpi.com/article/10.3390/genes17091039/s1.

## Abbreviations

MDAs	Müllerian duct anomalies
WES	Whole-exome sequencing
PAX8	Paired box protein Pax-8
MRKH	Mayer–Rokitansky–Küster–Hauser
AK5	Adenylate kinase 5
RCBTB2	RCC1 and BTB domain-containing protein 2
VCUAM	Vagina, cervix, uterus, and adnexa-associated malformation
ESHRE/ESGE	European Society of Human Reproduction and Embryology/European Society for Gynaecological Endoscopy
ASRM MAC2021	American Society for Reproductive Medicine Müllerian Anomalies Classification 2021
DEGs	Differentially expressed genes
PCA	Principal component analysis
qPCR	Quantitative real-time PCR

## Appendix A

**Table A1:** Primers used for Sanger sequencing of patients with the PAX8 variant.

Primer Name	Sequence (5′→3′)
p.L264P-F	CAACAGTGTGGTGAAACCAGGAA
p.L264P-R	GCCCGCCTCTCCTCTCCA

**Table A2:** Primers used in RT-qPCR.

Primer Name	Sequence (5′→3′)
qPCR-ACTB-F	GCACAGAGCCTCGCCTT
qPCR-ACTB-R	GTTGTCGACGACGAGCG
qPCR-PAX8-F	ATCCGGCCTGGAGTGATAGG
qPCR-PAX8-R	TGGCGTTTGTAGTCCCCAATC
qPCR-AK5-F	TCTAAGCCCGAAGATCCAGTAG
qPCR-AK5-R	GTGACTGTCCTCCATTTAGTGG
qPCR-RCBTB2-F	CAAGCCAGTACAGGCTACTCT
qPCR-RCBTB2-R	CCGAGGTTCAATGGTGCTCTG
qPCR-IFT88-F	GGGTCCAAGACATCTCTGGC
qPCR-IFT88-R	CATGGGTCTAGTAACTCCATCCT
